# Structural barriers to HIV prevention among men who have sex with men (MSM) in Vietnam: Diversity, stigma, and healthcare access

**DOI:** 10.1371/journal.pone.0195000

**Published:** 2018-04-03

**Authors:** Morgan M. Philbin, Jennifer S. Hirsch, Patrick A. Wilson, An Thanh Ly, Le Minh Giang, Richard G. Parker

**Affiliations:** 1 Department of Sociomedical Sciences, Mailman School of Public Health, Columbia University, New York, New York, United States of America; 2 Center for Research and Training on HIV/AIDS (CREATA), Hanoi Medical University, Hanoi, Vietnam; University of Toronto, CANADA

## Abstract

Men who have sex with men (MSM) in Vietnam experience disproportionate rates of HIV infection. To advance understanding of how structural barriers may shape their engagement with HIV prevention services, we draw on 32 in-depth interviews and four focus groups (n = 31) conducted with MSM in Hanoi between October 2015- March 2016. Three primary factors emerged: (1) Diversity, both in relation to identity and income; Vietnamese MSM described themselves as segregated into Bóng kín (hidden, often heterosexually-identified MSM) and Bóng lộ (‘out,’ transgender, or effeminate MSM). Lower-income, ‘hidden’ MSM from rural areas were reluctant to access MSM-targeted services; (2) Stigma: MSM reported being stigmatized by the healthcare system, family, and other MSM; and (3) Healthcare access: this was limited due to economic barriers and lack of MSM-friendly services. Our research suggests the need for multiple strategies to reach diverse types of MSM as well as to address barriers in access to health services such as stigma and costs. While a great deal has been written about the diversity of MSM in relation to gender performance and sexual identities, our research points to the substantial structural-level barriers that must be addressed in order to achieve meaningful and effective HIV prevention for MSM worldwide.

## Introduction

HIV prevalence among men who have sex with men (MSM) in Asia is greater than 10% among certain communities [1–3], and recent research suggests emergent epidemics in countries as varied as Thailand [4], India [5], and the Philippines [6]. This includes MSM in Vietnam who are in urgent need of effective HIV prevention. Since 2011, when HIV sentinel surveillance was first introduced in Vietnam, HIV prevalence among MSM has increased steadily, reaching a national prevalence of about 5% in 2015 [7]. In Vietnam’s two largest urban settings, Hanoi and Ho Chi Minh City, a 2015 HIV sentinel surveillance survey estimated MSM’s HIV prevalence at 12% [8–9,10]. These rising HIV rates among MSM are shifting the epidemic from one driven primarily by unsafe injection to one also driven by unsafe sex [11]. Indeed, in 2012 the Vietnamese government indicated that MSM (by which they meant MSM and transwomen) were the only groups in Vietnam for whom incidence was projected to increase [11].

Despite rising rates of HIV infection, studies show that MSM in Vietnam have limited access to basic HIV services including prevention [12][13] and testing [14]. Among HIV positive MSM in Vietnam, retention in HIV services and adherence to antiretroviral therapy (ART) is poor [14]. This is consistent with MSM’s poor access to healthcare more generally [15,16], which has been linked to economic barriers and difficulties identifying providers with whom MSM feel comfortable [17,18].

Facilitated by large increases in international resources and support, Vietnam has seen a rapid scale up of HIV treatment and prevention services over the past two decades [19]. HIV/AIDS is now one of the one of the country’s best-funded public health priorities. The number of people on ART has increased 100-fold, from 500 in the early 2000s to 50,000 by 2010 [19]. HIV prevention programs have been scaled up across the country; by 2012, free condoms were distributed for injection drug users (IDU) and female sex workers (FSW) across all provinces, and needle exchange was implemented in 88% of provinces [20]. However, targeted prevention services for MSM remain limited in urban settings, much less in rural settings [17]. As of 2017, two pre-exposure prophylaxis (PrEP) pilot demonstration projects had been launched in Vietnam [21], though PrEP has yet to be approved for large-scale implementation.

Few studies have examined barriers to HIV prevention services in Vietnam, specifically for MSM. To date, most HIV research in Vietnam has been conducted among either drug users or People Living With HIV/AIDS (PLWHA) [22]. Among drug users, punitive drug policies, stigma and discrimination have been identified as structural barriers to HIV prevention and treatment [23][24]. Among PLWHA, stigma, lack of patient confidentiality, lack of social support, and a shortage of HIV/AIDS specialists have been identified as additional barriers to HIV care [25,26]. MSM may experience additional unique barriers to HIV prevention owing to stigma and discrimination against homosexuality, which continues to be widespread in Vietnam [27,28]. An additional and under-explored barrier relates to the failure of existing public health programs to recognize and account for diversity among MSM. In fact, across public media and policy, MSM and other sexual minority populations are often misrecognized [29] as ‘merely’ imitating Western fashion, rather than being ‘truly’ gay or bisexual [28]. In Vietnam, the most common distinction among MSM is between *bóng kín* and *bóng lộ* [27,30,31]. Bóng kín (*hidden gay/shadow*) refers to MSM who maintain a masculine gender performance and who publically present as heterosexual. Bóng lộ refers to MSM whose presentation is more effeminate, and who do not hide their sexual preference for men (*conspicuous shadow*); transgender women are frequently included in this category. Other studies have documented more than 24 local terms that denote different identities that homosexually active men might assume, depending on both individual preferences and contextual characteristics of male-to-male sexual exchange [31,32]. In Vietnam, it is unclear whether and how such diversity may constitute a barrier to engagement in HIV services, though studies conducted among MSM elsewhere in Asia suggest that a failure to account for such diversity in HIV prevention can undermine access and engagement [33]. Although the response to HIV has expanded healthcare access, visibility, and some rights for MSM, it has also entrenched the association between MSM and HIV in the public consciousness, which continues to bear substantial stigma [27,28]. Moreover, while it is more common for research on HIV and MSM to examine questions of diversity in relation to sexual identity (e.g., whether MSM do or do not identify as gay; if they do not identify as gay, how do they identify [34,35] and what is their gender performance [33,36]), there has been relatively little examination more generally of how MSM map across other axes of social stratification, and how other dimensions of social inequality shape the possibilities for engagement with HIV prevention.

To be sure, three decades of social science research on HIV prevention point more generally to the structural barriers that constrain the adoption of health-enhancing practices and the cultural factors that shape people’s preferences for practices with detrimental health consequences [37,38]. But to date, HIV research among MSM in Vietnam has paid little attention to the structural level, including social, cultural, political and economic factors. This paper focuses on three key structural issues—diversity, stigma and access to health care services–in order to lay the groundwork for–or raise questions that would be crucial to consider for—the successful implementation of HIV prevention among MSM in Vietnam and elsewhere in the region.

## Methods

Data collection in Hanoi, Vietnam occurred from October 2015- March 2016 as part of a project that explored HIV prevention and empowerment among men; this analysis included the MSM from that sample. This portion of the study aimed to assess structural factors that might shape men’s engagement with HIV prevention. This sample of MSM, who varied by age, income, birthplace, and sexuality, included 32 in-depth interviews and four focus groups with 31 MSM. The focus groups were divided by identity (i.e., bóng lộ versus bóng kín) and age (18–29 and over 29). Of the four groups, one consisted of bóng lộ ages 18–29 (n = 8), one of bóng lộ ages 30-plus (n = 8), one of bóng kín ages 18–29 (n = 7) and another of bóng kín ages 30-plus (n = 8).

The study utilized both in-depth interviews and focus groups to optimize discussion of sensitive topics, since relative to one-on-one interviews, researchers have described focus groups as both enabling and constraining for facilitating conversations about sexual behavior and HIV prevention [39]. In this study, the goal of conducting focus groups was to facilitate a more open discussion by shifting power from the interviewer to the participants and thus giving participants greater influence over the direction of the conversation [40].

For both focus groups and in-depth interviews, men were recruited by health educators from a university-based HIV clinic, all of whom were MSM. Recruitment occurred through posters and fliers posted at health clinics, meetings and community events, and public and hidden Facebook groups and smartphone apps that cater to MSM. Upon recruitment, men were asked if they would prefer to participate in the focus group or in-depth interview; in-depth interview participants were also allowed to choose if they wanted to be interviewed by a male or female researcher. The interviews and focus groups were conducted by two trained, Master’s level interviewers with experience working in public health, HIV and with MSM. In-depth interviews lasted approximately 100 minutes and focus groups lasted around 120 minutes. Topics included family, educational and social history, experiences with stigma and discrimination, and engagement with medical care and HIV prevention-related services and programs. Interviews and focus groups occurred in a private room at the university-based HIV clinic, and men received 200,000 VND (~$10) to compensate for their time. Data collection also involved participant observation in locations frequented by these men such as bars, parks, and clubs. All participants provided written informed consent. The Institutional Review Boards at Columbia University Medical Center and Hanoi Medical University approved all aspects of this study.

All data were digitally recorded, transcribed verbatim, and uploaded into Atlas.ti; relevant sections were translated. We used theories of gender, health and sexuality to develop initial codes that were relevant to our research questions; we also examined the interview and focus group guides to develop salient codes. Two researchers then read through the transcripts to conduct line-by-line coding to identify salient themes. The study team developed a codebook that included overarching themes such as stigma, gender and sexuality, engagement in care, social support, and HIV prevention-specific codes. To analyze the data, we looked both within and across cases to better understand how the lived experience of homosexuality in Vietnam might affect men’s desire for, and use of, HIV prevention. The analysis for this paper explored the community- and structural-level factors that would influence MSM’s ability to engage with HIV prevention. The critical optic that we brought to these questions was informed both by our longstanding research on sexuality and HIV in Vietnam [41] and by parallel work that we had conducted examining social barriers to Black MSM’s engagement with HIV prevention in New York City [42].

## Results

We recruited nearly equal numbers of Bóng kín and bóng lộ for the interviews and focus groups; nearly one-third of the bóng lộ identified as transgender (Table 1). Participants ranged in age from 18–58, and the majority came from rural areas where homosexuality-related stigma was particularly high. Most reported migrating to Hanoi to find jobs, pursue higher education, and extend their social networks. The majority reported working in the informal sector (e.g., as street vendors or performers). Three primary factors emerged that might influence MSM’s ability to engage with HIV prevention: diversity, stigma, and healthcare access.

**Table 1:** Demographics of the MSM from the in-depth interviews and focus groups.

**In-depth interview participants (Total: 32)**			
**Category**	**Age**	**Participants (n)**	**Total**
*Bóng kín* *(Hidden gay/shadow)*	18–29	10	17
	30+	7	
*Bóng lộ* *(Conspicuous shadow)*	18–29	9	15
	30+	6	
**Focus group discussion participants (Total: 31)**			
*Bóng kín* *(Hidden gay/shadow)*	18–29	7	15
	30+	8	
*Bóng lộ* *(Conspicuous shadow)*	18–29	8	16
	30+	8	

### A. Diversity

Participants were asked to discuss the different types of MSM in Vietnam. Many immediately expressed a dislike of the term “MSM community” and saw it as a term employed by researchers and government-sponsored programs to create an illusion of uniformity among what was, in fact, a quite fractured group. Since the term “MSM community” is used frequently in public health programming and research in Vietnam, the interviewers raised it in order to generate discussion about how MSM feel about this term, and what other terms they might prefer. Respondents depicted MSM in Vietnam as stratified by several key axes: sexual identity, gender performance, socioeconomic status, and age. Many participants reported not identifying with the ‘MSM community’ and instead relied on their own smaller group of long-term and trusted MSM.

#### Gender/Sexuality

The majority who identified as *bóng lộ* described themselves as effeminate men or transwomen, who were ‘out’ and unafraid to display their sexuality; the category of *bóng lộ* encompasses both MSM and transgender women. The most relevant axis of gender was not identity but performance and how ‘out’ a person acted in front of others. The remaining MSM in our sample considered themselves to be *bóng kín* who were described as hidden, often heterosexually-identified MSM, who frequently criticized other MSM as ‘conspicuously gay’. *Bóng kín* reported smaller networks, were hesitant to participate in MSM-targeted activities and frequently self-segregated because they were afraid of being ‘outed’ by *bóng lộ* who might appear more obviously gay or effeminate. A 27-year-old bóng lộ (IDI_LO_09) noted that, “*White-collar MSM or married MSM (i*.*e*., bóng kín) *usually hang out together in a small group; It’s easier for them to conceal it [sleeping with men] because their family and other people will never doubt their sexual identity*.*”* He also described additional types of segregation: “*Moreover*, *both masculine and feminine gays are usually hesitant to hang out with transgender women or transvestites*. *They are afraid that it will draw people’s attention*.*”*

Importantly, *bóng kín* described themselves at low risk for HIV because they did not engage in sex with a wide range of MSM as did *bóng lộ*; other MSM felt that *bóng kín*’s attempt to hide their sexuality actually put them at higher risk (e.g., because they paid for sex and engaged in casual sex in places like bars and saunas). In addition, *bóng lộ*’s more overt displays of sexuality afforded them greater access to HIV-focused campaigns and programming.

#### Socioeconomic status (SES)

Socioeconomic status and access to resources were other important axes of diversity. Economically disadvantaged MSM, especially *bóng lộ* who lacked the means to appear more ‘beautiful’, felt disenfranchised and were less likely to engage with the broader MSM community. As a result, lower-income MSM, often from rural areas, were reluctant to access MSM-targeted services. One respondent described the importance of consumption and beauty:

Money and beauty are the two biggest issues for MSM… MSM who are both good-looking and rich usually make friends with those as rich and good-looking as they are. Beauty is a big deal too. It’s common that a gay man will not reply to your message if he doesn’t like your look. And you never find a group that has both good-looking and not-good-looking MSM

(26, bóng lộ; IDI_LO_07).

These descriptions of socioeconomic status and beauty were often also intertwined with age. This, in turn, was an axis of diversity as it impacted men’s social networks and the type of information to which men had access.

#### Age

Men uniformly described how there was very little social mixing between older and younger groups of MSM, as one man noted:

There is a saying: Birds of a feather flock together…It is the same for us. Those, who are the same age hang out together. For example, 19, 20-year-old gays do not hangout with us [middle age gays]; they are friends with each other. We are getting old, older than them, so we find people our own age to be friends with. Because our lifestyle, our thinking is different than theirs, so we cannot be in a same group

(58, bóng lộ; IDI_LO_06).

One individual noted that younger MSM often spent time with each other because they lacked the resources to engage with older men, *“for example*, *a student will not talk to older MSM who look richer or more beautiful than him because MSM usually make friends with men who are similar to them”* (26, bóng lộ; IDI_LO_07). In addition to not mixing socially, age mattered in how men understood labels like MSM, and how they engaged with other MSM. Older MSM grew up with limited Internet, which meant that their understanding of homosexuality-related norms came almost exclusively from friends, family, and Vietnamese society. In contrast, younger MSM frequently used the Internet to read information that was supportive of homosexuality and informative of other countries’ more accepting approaches. Men repeatedly described how the younger MSM were different than the older, more hidden and shy, generation. One man noted, *“Now*, *homosexuality is more acceptable and understandable than how it was in the past*. *It is more open*, *so youth are willing to be “conspicuous” to family*, *friends and others”* (30, bóng lộ; FGD_LO_2). In addition, whereas younger men accessed much of their HIV-related information through higher levels of engagement with MSM-focused outreach programs, older men often reported poorer access. The majority of HIV prevention research and services is targeted to specific types of MSM and these groups, *“organize events exclusively for those who identify as LGBT*, *or for MSM only”* (25, bóng lộ; IDI_LO_17). This means that men who openly identify as MSM and engage with various NGOs are included in HIV prevention research, whereas older men are often not included. Even the name of these groups (e.g., ‘Youth Dream’) focus on a younger demographic. This means that many MSM—particularly older MSM—might not be aware of existing services—or even see themselves as in need of services—that might offer HIV prevention.

### B. Stigma

MSM described intersecting and multi-layered stigma that came from other MSM, family and friends, and in professional settings. As a result, men reported a lack of family and social support, the need to work consistently to hide their sexuality, and persistent mental health issues (e.g., loneliness, emotional deprivation). Despite describing wide social networks, they also frequently reported having only a limited number of close friends upon whom they could rely.

*Bóng lộ* reported experiencing the most stigma and facing high rates of discrimination, unemployment, health issues, and financial hardship. *Bóng lộ’s* overt displays of gender/sexuality even generated stigma from within the MSM community because they felt that such behavior challenged society’s ability to accept gay people as a whole. As one man noted, “*hidden gays really stigmatize the more obviously gay guys*. *Because they wonder why a homosexual man has to be forward and flamboyant in such a way that makes other people hate them*, *and look down on the gay community*. *Mostly*, *bóng kín stigmatize ‘bóng lộ gays”* (24, *Bóng kín*; IDI_Kin_03) In addition, there was a large amount of discrimination between men who identified as gay and the transgender community: “*Within the MSM community*, *there are a number of gays who don’t like transgender individuals*. *I don’t know why*. *They just say that they don’t like them*. *That’s all”* (29 bóng lộ; FGD_LO_01).

*Bóng kín* felt additional pressure to remain hidden because they received societal privileges that came with assumed heterosexuality. This pressure caused high levels of psychological stress:

I always think of how to hide it so people will not find out who I really am. I feel anxious and nervous all the time. For example, when homosexual things come up in a conversation, my college friends will make ugly, stigmatizing comments. And I either keep silent or partake to be like them so they will never doubt [my sexuality]

(21, bóng kín; IDI_Kin_13).

#### Family stigma

The majority of participants chose not to disclose to their families because they anticipated a negative response such as being locked up, beaten, or losing financial support. One man described that when his father found out:

“He shouted at us: What the hell are you doing? How can you do such sick stuff?” He cursed us, despised us, but kept calm, and did not beat us. My two sisters and I were crying a lot, and they fought with my father to protect me. But my father said that it was a disease; and that the support my sisters gave me would kill me instead of helping me get better

(24, bóng kín; IDI_Kin_03).

As a result, many MSM moved to urban centers, though becoming fully independent was harder for younger men who had to manage familial expectations around their sexuality and future reproductive potential while still relying on their families financially. This dependence limited their ability to leave home and forced them to conceal their sexuality in ways demanded by families. When asked why he did not come out in his hometown one man said:

If people from home know that I’m gay, they will not speak to me and they will spread gossip about me and my family will hear it. They might say something like: ‘oh gosh, that is the son of the X family, he loves boys.’ That pressure is so tough that I’m not sure that I can handle it, so I’m not willing to come out

(19, bóng lộ; IDI_LO_04).

Even MSM who were accepted by some members of their families (e.g., siblings but not parents) reported an internalized homophobia and feelings of inadequacy for not fulfilling society’s masculine norms. MSM described having to choose between maintaining appearances (i.e., married with children) while keeping their sexuality hidden, or living with another man but feeling guilty about not fulfilling familial expectations and having to continuously explain why he was not yet married with children.

#### Stigma at school/work

Some bóng kín described employment as police officers, military officers, or politicians, and reported that they could never reveal their sexuality because it would damage their career. In contrast, bóng lộ reported that their more overt displays of sexuality often caused them to drop out of school, which complicated their ability to find legitimate and stable employment. One man described what would happen if a bóng lộ, particularly a transgender woman, applied for a job, “*they will never get the job*. *You see*, *they look like a woman*, *but their ID card is different [a man’s ID]*. *People will never hire them…Their life is tough*, *they face a lot of stigma”* (22, bóng lộ; IDI_LO_01). As a result, some individuals began engaging in sex work to earn money.

The MSM in this study described sex workers as a particularly vulnerable subgroup due to intense stigma, unmet medical needs and high levels of mobility. Sex workers consisted mostly of young men with limited education who hail from rural areas and who had come to the city to earn money, pursue an education, and avoid stigma at home. One man described how, “*everything will be ok if no one knows that he sells sex*, *but if someone finds out what he does for a living*, *they will gossip about it*, *and call him a pervert*, *full of depravity*, *something like that”* (19, bóng lộ; IDI_LO_04). Another man added that male sex workers are doubly stigmatized because they sell sex and they sell it to men: “*And even we*, *the MSM community*, *stigmatize them*, *and despise them for what they do for a living*. *I think that they suffer from stigma more than us and more than women who sell sex”* (22, bóng lộ; FGD_LO_01). Stigma from other MSM also existed because they felt that men only engaged in sex work because of their high sex drive and desire for more money, rather than due to any social-structural constraints. The extremely stigmatized nature of same-sex sexual practices also present barriers to service access and social support; consequently, they may also limit MSM’s willingness to engage with prevention and to present for regular HIV testing.

### C. Health service-level barriers

#### Reluctance to disclose same-sex behaviors in clinical settings

Respondents frequently noted that they did not want to disclose their same-sex behaviors, or sexual identity, to a clinician because they were afraid of the potential for stigma and discrimination. In addition, they worried that such a disclosure would impact the quality of care that they would receive, and described barriers related to cost and the lack of MSM-friendly services. This was particularly true for sexually transmitted infection (STI)-related treatments, which often forced a disclosure of sexuality. Men reported that they therefore often delayed treatment until the symptoms were obvious. One man told us that:

They don’t get treatment at all. I’ve met guys who told me that their anus was swollen. Then, I asked them about treatment, but they said that they were afraid of going to see a physician, of going to the hospital…I think that they are afraid of telling a physician why they have such symptoms. They are afraid that physicians will be negative while providing treatment if they know the man is gay

(22, bóng lộ; IDI_LO_14).

This anticipated stigma had a direct impact on access to treatment as well as the type of treatment men received because it limited their ability to be honest about potential risk factors. One man shared how his friend’s failure to disclose meant that he wasn’t treated correctly:

My friend had anal sex for the first time and it hurt so bad that he had to go to hospital. A clinician wanted to conduct an examination but he wouldn’t let the clinician do that. So, the clinician could not make a diagnosis and just prescribed a painkiller. I think that it’s a barrier for MSM because they are reluctant to disclose to a heterosexual person that they just had sex with a man or that they’re gay. The more they conceal it, the more serious their disease gets

(26, bóng lộ; IDI_LO_07).

#### Cost

Because of social discrimination and labor market exclusion, Vietnamese MSM often faced economic barriers to healthcare access. Shifts in the healthcare system’s structure require increasing out of pocket costs, and men were frequently unable to afford such costs, especially to pay for services that did not meet their needs. One man shared how: *“They are reluctant to see doctors and only go when disease symptoms are getting worse*. *And even after being diagnosed*, *they are not willing to receive treatment because the cost of treatment is too expensive”* (22, bóng lộ; IDI_LO_10).

Another told us how:

I took a gay friend to a clinic, and he was diagnosed with HPV and wanted to get treatment but could not afford to pay [for] it (~$45 US). And he did not dare to ask his parents for that amount of money. They would question and think that oh, you must be gay to get that disease

(22, bóng lộ; FGD_LO_01).

This demonstrates how the cost for treating an STI was a particular barrier for young MSM who were still financially dependent on their family, to whom they had often not disclosed.

#### Lack of tailored services

Individuals reported few MSM-friendly clinical services, and noted that many had closed recently due to budgetary constraints—a situation further exacerbated due to the withdrawal of the Presidential Emergency Plan for AIDS Relief (PEPFAR) from Vietnam [43]. This forced men to access care through traditional clinics, and thus risk the aforementioned high levels of stigma. Men noted explicitly how the lack of MSM-friendly services limited access to health information and treatment because they only went to clinics if symptoms were quite severe; they never went simply to access information. One man reported:

There are many challenges that MSM face. First is limited access to health information, sexual risk and harm reduction. Because there is no place for MSM to come and share this kind of information […] I know one organization that ran for a long time, but I have not heard anything about it in the last few years

(26, bóng lộ; IDI_LO_07).

Administrative processes in clinic and hospital settings also limited MSM’s access to the healthcare system. This was particularly true for transgender individuals whose appearance did not match their identification card or medical records. One man shared how a transgender friend injected herself with hormones and went into shock. As a result, “*we took her to the emergency room*. *The next morning*, *the clinician asked for her ID and other documents*. *And she freaked out because she looked totally different than the picture on her ID*, *so she ran away from the hospital”* (22, bóng lộ; FGD_LO_01). This lack of sensitivity to the needs of MSM and transwomen also exists on a structural level, specifically regarding the availability of risk-reduction measures. For example, it is very difficult to access lubricant in Vietnam, or even lubricated condoms. As one man noted:

They need lubricant, but pharmacies often sell condoms but no lubricant. And the condoms they sell are non-lubricated condoms, and using that kind of condom makes men feel unreal and hurt. So, they decide to not use it, and they are easily infected with STIs

(22, bóng lộ; IDI_LO_10).

In addition, the few men who spoke openly with their providers about their sexual history reported that providers often knew little about their specific needs and were unable to suggest HIV prevention options beyond condoms. The MSM we spoke with reported numerous barriers to accessing health-related services, which would also limit their ability to access HIV prevention and also basic healthcare services more generally. Even the men who could afford to visit a provider might not fully benefit from the visit if they feel unable or unwilling to disclose that they sleep with other men. This means that the provider will be less likely to discuss HIV prevention.

## Discussion

While previous studies have emphasized resource limitations and low awareness of PrEP as challenges to PrEP scale up in Vietnam and elsewhere across the region [44–46], our findings revealed three primary factors that may shape how MSM in Vietnam engage with HIV prevention: diversity among MSM, multi-layered and multi-level stigma, and low access to primary healthcare services. Data collection occurred only in Hanoi and men’s experiences might therefore not reflect those of more rural areas. However, given that men described Hanoi as one of the most accepting places in Vietnam, the stigma and barriers described here are likely to be even more severe elsewhere.

Vietnamese MSM reported numerous sub-groups and internal divisions. This aligns with previous research that demonstrated divisions among MSM due to age, socio-economic status, and hometown [27,30,31]. Young MSM, for example, struggled to integrate social norms around sexuality with the more inclusive messages they found online. They also reported frequent Internet use, and the ability to navigate information found online. This aligns with U.S.-based research that demonstrates how the Internet, social media, and text messaging can be used to disseminate HIV-related and PrEP-focused messaging for young people [47–49]. Older MSM, in contrast, reported minimal Internet use and a frequent distrust of information found online. In particular for Vietnam, whereas *bóng lộ* were likely to mention some involvement with community organizations, bóng kín reported little engagement with MSM-focused outreach and groups. In addition, *bóng kín* did not view their behaviors as particularly risky and therefore did not feel a need to engage in HIV prevention. As documented in other Asian contexts [50], diversity among MSM presents a challenge for HIV prevention. In particular, *bóng kín* may be unlikely to access HIV-related services that target sexual minorities. Instead, this group might be better targeted for HIV prevention through more general messaging (e.g., around masculinity and health) that does not assume a non-heterosexual identity [51,52]. These findings demonstrate the complexity of how MSM identify across different spaces and groups, and what that might mean for tailoring HIV prevention-related outreach and interventions. Messaging will need to be targeted and varied in order to ensure that MSM who vary by factors above and beyond sexual identity and gender—such as age and SES—receive information that is relevant to their lives (Table 2).

**Table 2:** Questions to consider when scaling-up HIV prevention for MSM.

*Themes Relevant to HIV prevention*	*Questions to consider for HIV prevention among MSM*
**Diversity within MSM**	What are the local categories for MSM and who might not fit into those categories?
	Which types of MSM are targeted in HIV prevention interventions and who might therefore be missed by current HIV prevention efforts?
	What are the most socially consequential axes of diversity within the MSM being targeted (e.g., SES, age, social groups, identity)?
	Within these diverse groups of MSM, who might face particular challenges in accessing primary care?
	How might demographic factors (e.g., age and SES) impact HIV- and prevention-related knowledge and willingness to access relevant prevention methods?
**Stigma**	How might stigma impact the ways MSM present their gender and sexuality (e.g., how ‘out’ they are) and their willingness to engage in HIV prevention interventions?
	How might MSM’s reluctance to disclose same sex behaviors as a result of stigma impact the ways that they access HIV- and prevention-related information?
	Many MSM face systematic economic discrimination and are relegated to low-paying and unstable employment. How can biomedical HIV prevention modalities (e.g., PrEP) be scaled up in ways that address structural-level stigma and facilitate such men’s access and ability to remain adherent?
**Health Service-level Barriers**	How can providers be trained to ensure that they are providing the most up-to-date and accurate prevention-related information in a way that is supportive and inclusive of MSM?
	Since many MSM lack a primary care provider, what are other ways that they might learn about and access prevention?
	How might the intersection of employment and health insurance impact men’s access to healthcare?

The last decade has also coincided with the rapid emergence of increasingly visible communities of MSM and transwomen in larger metropolitan areas like Ho Chi Minh City and Hanoi [41]. Some cosmopolitan young men have begun to assume an ‘out and gay’ identity because the assertion of this identity is viewed a marker of social class and modernity. Vietnamese community-based organizations (CBOs) that have only recently begun to respond to the HIV epidemic among MSM have relied primarily on peer outreach workers who are openly gay men or effeminate MSM. In addition, CBOs are relatively rare outside the larger cities, which limits their ability to access rural MSM. If the Vietnamese civil society response follows its current trajectory, it will do so at the expense of *bóng kín*, closeted men and gender-nonconforming individuals [53,54].

Consistent with studies conducted in other Asian contexts [55], the impact of stigma permeated MSM’s lives. It caused them to leave home, drop out of school, choose jobs with less stability and lower pay, and limited their willingness and ability to seek care. Consistent with other research about segregation between bóng kín and bóng lộ [27,30,31], bóng lộ reported higher levels of stigma and social exclusion. As Oldenburg et al (2016) have argued, combination HIV prevention interventions that include a behavioral component that addresses stigma, coupled with structural-level interventions, may have the greatest impact on preventing HIV transmission among MSM, particularly bóng lộ [21].

Stigma strongly influenced healthcare seeking and STI testing among MSM. Providers often only recommend HIV testing when a man presented with STI symptoms, but MSM often avoid clinics due to stigma [50,55–57]. Similar to studies in the U.S. [56,57], even the Vietnamese men who did present for care were often hesitant to disclose their sexuality or sexual practices, which affects clinicians’ likelihood of accurately assessing individuals’ healthcare needs to determine which forms of HIV prevention methods may be most appropriate for them [17]. Even MSM who can and do access healthcare often face stigma from healthcare providers who are frequently non-responsive to their unique needs.

Access to combination prevention in Asia remains limited and in Vietnam it continues to vary considerably by geography: larger cities (e.g., Hanoi, Ho Chi Minh City, and Hai Phong) often have CBOs that can provide condoms and lubricant, peer education and outreach, and confirmation testing and linkage to care; rural areas have very few services [45]. Even with free HIV testing and attempts to target MSM, there has not been a substantial reduction in HIV incidence among MSM in Vietnam [7]. As a result, advocates and researchers have called for the scale up of PrEP, which is not yet approved in Vietnam. PrEP is currently only available through two demonstration projects in Ho Chi Minh City, which have enrolled about 1,000 participants and ended in early 2018. While the Vietnamese government has committed to implementing PrEP, there has been no concrete financial investment, which means that even if PrEP were approved, it is unclear whether it would be accessible to the men like those interviewed as part of this study. Resource limitations remain a significant challenge to PrEP scale up in Vietnam and across the region [44,46]. Specifically, it is unclear whether PrEP would be covered by health insurance since preventive measures are rarely covered in Vietnam.

The healthcare system’s fragmentation forms a critical part of the context for understanding healthcare access among this economically vulnerable and socially marginalized group. The increased visibility of MSM has coincided with the dissolution of the Vietnamese safety net and the emergence of parallel public and private health systems, leading to increases in out of pocket expenses and additional barriers to care [58]. Overall the health system is characterized by poor continuity of care, weak coordination between public-private facilities [59,60], a lack of public trust in publicly-funded primary care, and extreme overcrowding in tertiary care facilities. The privatization of healthcare has occurred in many countries over the last decade [61,62], suggesting that this finding regarding the fraying safety net and weakening HIV prevention is relevant across multiple geographic locales.

As a result, inequalities in access to, and utilization of, healthcare have increased [15,16]. In 2010, only 37% of people in the poorest quintile received any medical care in the last year compared to 45% amongst the richest quintile [60]. Disparities were particularly stark for MSM who often lacked money to access care or, when they could, were unable to find providers with whom they felt comfortable [17,18]. Bóng lộ in particular reported an awareness of their HIV/STI risk but, along with young MSM, reported limited financial resources [47,48].

### Strengths and limitations

We explored the multi-level barriers to health and wellness that MSM face, and the implications of those barriers for HIV prevention. Though data collection occurred only in Hanoi, many of the men had migrated from rural areas and so could speak to experiences in both rural and urban locales. In addition, given that men described Hanoi as one of the more accepting places in Vietnam, the stigma and barriers described here are likely even more intense than in other locales. Data collection included both interviews and focus groups, which allowed us to access a wide range of experiences and hear about both individual experiences and group norms. While focus groups may have impacted the comfort and candor of participants, we segmented the groups by age and sexual identity in order to limit this phenomenon—we also allowed men to choose whether to participate in an interview or focus group. In addition, similar data emerged in the interviews and focus groups suggesting that men shared their experiences and concerns.

## Conclusions

Vietnam provides an example of the substantial structural-level barriers that must be addressed in order to achieve meaningful and effective HIV prevention for MSM worldwide. More generally, global expansion of HIV prevention including new methods such as PrEP among MSM has focused primarily on individual-level factors and little has been said about how structural-level challenges such as stigma, healthcare access, and diversity within MSM impact access to and engagement with HIV prevention. Our study has several implications for the scale-up of HIV prevention among MSM. Findings demonstrate the need to address structural-level barriers (e.g., around MSM-friendly clinics, cost-related barriers, and stigma) before HIV prevention can be successful. As HIV prevention is scaled-up in Vietnam, these findings provide key insights about the need to tailor messaging in ways that will resonate with different groups of MSM, and also demonstrate the importance of additional research to better understand how the healthcare system can be adapted to serve sexual and gender minorities. This study focused on the views of *bóng kín* and *bóng lộ* irrespective of their attachment to the gay community. Future research is needed to examine how the growing and increasingly visible LGBT community in Vietnam might be able to support HIV prevention. Our findings, and the questions that we raise in Table 2, are of broad relevance to those working in HIV prevention among MSM anywhere; casting our eyes back over three decades of the HIV epidemic in the U.S., it is apparent how stigma regarding gender performance and many other axes of social stratification functioned to condense HIV among more socially vulnerable groups of MSM, even as the epidemic has become relatively contained among more privileged White gay men [63]. Those in the HIV prevention community would do well to learn a lesson from the history of the U.S. epidemic, and build prevention programs that mitigate inequality rather than amplifying it.
